# Safety and efficacy of switching from low molecular weight heparin to dabigatran in patients undergoing elective total hip or knee replacement surgery

**DOI:** 10.1186/s12959-015-0066-9

**Published:** 2015-11-26

**Authors:** Christian Wurnig, Andreas Clemens, Helmuth Rauscher, Eva Kleine, Martin Feuring, Reinhard Windhager, Josef Grohs

**Affiliations:** Orthopaedisches Spital, Speisinger Strasse 109, 1130 Vienna, Austria; Center for Thrombosis and Hemostasis, University Medical Center Mainz, Mainz, Germany; Boehringer Ingelheim Pharma GmbH & Co. KG, Ingelheim am Rhein, Germany; Department of Orthopaedics, Medical University Vienna, Vienna, Austria

**Keywords:** Dabigatran, Low molecular weight heparin, Switching, Hip replacement surgery, Knee replacement surgery, Thromboprophylaxis

## Abstract

**Background:**

The aim of this study was to assess the safety and efficacy of switching therapy from low molecular weight heparin (LMWH; enoxaparin) to dabigatran for prevention of venous thromboembolic events (VTE) in patients undergoing elective total hip or knee replacement surgery (THR/TKR).

**Methods:**

This was a prospective, multicenter, open-label, single-arm, observational, study in patients undergoing THR or TKR who were to receive enoxaparin 40 mg for thromboprophylaxis. Enoxaparin was initiated before or after surgery according to local practice, and was switched to dabigatran 220 mg once daily at a time point chosen by the investigator. The coprimary endpoints were major bleeding events, and the composite of symptomatic VTE and all-cause mortality, from last use of enoxaparin to 24 h after last intake of dabigatran.

**Results:**

Altogether, 168 (81 THR, 87 TKR) patients were enrolled, of whom 161 received both enoxaparin and dabigatran, 2 received dabigatran only and 5 received enoxaparin only. The median time of the first dabigatran tablet was 24.0 h after the last LMWH dosage and the median number of days on dabigatran treatment was 36 days. No symptomatic VTE or death occurred during the study. One major bleeding event was seen at the surgical site and required treatment cessation. Three minor bleeding events were observed.

**Conclusions:**

In the normal clinical setting, switching from LMWH to dabigatran in patients who had undergone THR and TKR was safe and effective in preventing VTE. The reported adverse events and serious adverse events were consistent with the known safety profile for dabigatran. Switching from a subcutaneous to an oral anticoagulant may offer greater convenience in the outpatient setting after discharge.

**Trial registration:**

ClinicalTrials.gov identifier NCT01153698.

## Background

Without thromboprophylaxis, major orthopedic surgery, such as total hip or knee replacement (THR or TKR), carries a high risk of venous thromboembolic events (VTE), which manifests as deep vein thrombosis (DVT) in two-thirds and as pulmonary embolism (PE) in approximately one-third of patients [1]. This is because orthopedic surgery induces local and systemic thrombin generation, which can trigger thrombus formation and thrombotic events. Without thromboprophylaxis, venographic DVT can be observed in 40–80 % of patients [2]. The risk of nonfatal versus fatal PE for elective orthopedic surgery patients without thromboprophylaxis is 1.8–7.0 % and 0.2–0.7 %, respectively [3].

Effective anticoagulation significantly reduces the risk of VTE and is recommended in various international consensus guidelines [4, 5]. Therefore, the majority of patients receive some kind of thromboprophylaxis; low molecular weight heparin (LMWH) is commonly used in many countries in Europe [6, 7]. However, as many as 40 % of patients do not receive appropriate thromboprophylaxis as recommended by consensus guidelines mentioned above [8, 9]. The subcutaneous route of administration for LMWH presents as a barrier to applying the more effective extended prophylaxis regimens up to 35 days after THR, and many patients do not continue anticoagulant prophylaxis after discharge from hospital [10]. Bjørnarå BT, et al. showed that most cases of VTE after orthopedic surgery developed following hospital discharge, confirming the need for continued thromboprophylaxis [11]. Use of an oral therapy simplifies and optimizes posthospital management for these patients and enables continuation of thromboprophylactic therapy for the recommended time of up to 35 days [4, 12].

The non-vitamin K antagonist oral anticoagulant (NOAC), dabigatran etexilate (henceforth, “dabigatran”), is a reversible direct thrombin inhibitor, approved in many regions or countries including Europe, Canada, and Australia for this indication; it has recently been included in guidelines for the prevention of VTE in patients undergoing orthopedic surgery [4]. Dabigatran offers potential advantages over currently available anticoagulants. It eliminates the need for parenteral or subcutaneous administration, increasing compliance particularly when outpatient antithrombotic treatment is required after early hospital discharge.

After major orthopedic surgery, dabigatran has favorable, slow postoperative onset with delayed absorption and a reduced plasma peak concentration that does not tend to disturb the ongoing hemostatic process [13, 14]. The results from randomized phase 3 trials (RE-NOVATE®, RE-NOVATE™ II, and RE-MODEL™) in elective major orthopedic surgery showed that dabigatran (150 mg and 220 mg once daily [qd]) has similar efficacy compared with enoxaparin 40 mg qd in adults [15–18].

The optimal start of thromboprophylaxis with LMWH in orthopedic surgery remains unclear; even guidelines are vague in this respect [4, 12]. In Europe, many orthopedic departments start perioperative prophylaxis with LMWH between 2 h before and 6 h after surgery, whereas others start prophylaxis 12 h preoperatively [19, 20]. A perioperative start is apparently more effective, but this is counterbalanced by a marked increase in the risk of major bleeding in comparison with a preoperative or postoperative regimen [21]. Unlike LMWH, oral prophylaxis with dabigatran is recommended to be initiated postsurgery. Patients should have sufficient hemostasis before therapy is initiated, and the recommendation is to start dabigatran therapy with a half dose 1–4 h postsurgery [22, 23].

The primary objective of this trial was to assess the safety (major bleeding events) and efficacy (symptomatic VTE and all-cause mortality) of a switch from enoxaparin 40 mg qd to dabigatran 220 mg qd for prevention of VTE in patients undergoing elective THR or TKR. The doses and administration schedules selected for this observational study reflect the European standard clinical doses for primary VTE prevention in the orthopedic setting [4].

## Methods

This was a multicenter, open-label, prospective, single-arm, study conducted at 7 orthopedic centers in Austria. The Institutional Review Board (Ethikkommission der Medizinischen Universität Wien, Wien, Austria) granted approval. All patients provided written, informed consent. The study was monitored by an independent Data and Safety Monitoring Committee. This study was registered in the ClinicalTrials.gov database with the identifier number NCT01153698.

Eligible patients were men and women with ages ≥18 years, who had undergone elective primary THR or TKR, and were to receive LMWH (enoxaparin 40 mg) subcutaneously for thromboprophylaxis according to the Committee for Medicinal Products for Human Use label. This study was terminated prior to reaching the target sample size due to slower than anticipated recruitment.

The primary safety variable was major bleeding events (MBEs) from last application of enoxaparin until 24 h after last intake of dabigatran. MBEs were, as in phase 3 clinical trials, defined according to International Society on Thrombosis and Haemostasis criteria: a bleed was considered major if ≥1 of the following criteria was met: fatal bleeding; clinically overt bleeding in excess of what was expected and associated with ≥20 g/L (corresponds to 1.24 mmol/L) fall in hemoglobin in excess of what was expected; clinically overt bleeding in excess of what was expected and leading to transfusion of ≥2 units packed cells or whole blood in excess of what was expected; symptomatic documented retroperitoneal, intracranial, intraocular, or intraspinal bleeding; bleeding requiring treatment cessation or, bleeding leading to reoperation. The co-primary efficacy variable was the composite of documented symptomatic VTE and all-cause mortality from last application of parenteral anticoagulant until 24 h after last intake of dabigatran.

Patients started treatment with enoxaparin 40 mg qd according to local clinical practice. Treatment with enoxaparin was initiated before or after THR or TKR surgery at the clinical discretion of the investigator. The timepoint of the switch was chosen by the investigator, based on clinical judgment, as this was a noninterventional study [24]. If the switch was performed on the day after surgery or at any later point in time, dabigatran was to be initiated with 220 mg, and not before the next scheduled dose of the LMWH would have been due.

A total of 3 visits were to be scheduled. The first (baseline) visit was to occur preoperatively ≤7 days before surgery and was to include an assessment of creatinine clearance for a dabigatran dosing decision according to the Summary of Product Characteristics [23]. The second visit was to be at the time of discharge from hospital or 24–48 h after the end of dabigatran treatment (whichever came first). Visit 3 (follow-up visit) was only to be performed if discharge from hospital occurred before the end of dabigatran treatment, and was to occur 24–48 h after the end of dabigatran treatment.

Patients were treated under the same surgical conditions and techniques, according to hospital protocol. Any bleeding event (i.e., major and nonmajor), symptomatic DVT or PE events that occurred during the study period had to be documented in the study case report form.

Statistical analyses were performed on data from all patients treated, i.e., all patients who received ≥1 dose of enoxaparin or dabigatran. The total treatment period is defined as the period from the first application of enoxaparin 40 mg until 24 h after administration of the last dose of dabigatran.

Due to the observational nature of this cohort study, all analyses were descriptive in nature, including p-values and confidence intervals (CIs) from statistical methods used for exploratory purposes. For the analysis of the primary endpoint, MBEs, and the number and percentage of patients with MBEs were presented including 2-sided 95 % CI (exact interval by Pearson and Clopper).

## Results

A total of 168 patients were enrolled and treated with dabigatran and/or enoxaparin. All patients underwent either TKR (51.8 %) or THR (48.2 %) surgery. Surgery was completed in all patients as planned. Demographic and baseline characteristics are given in Table 1. Overall, 57.1 % of patients were female, and patients ranged in age from 28 to 84 (median, 62) years.

**Table 1:** Demographic data of all 168 patients included in the study

	THR	TKR	Total
Treated, n (%)	81 (100.0)	87 (100.0)	168 (100.0)
Gender, n (%)			
Male	40 (49.4)	32 (36.8)	72 (42.9)
Female	41 (50.6)	55 (63.2)	96 (57.1)
Age (years), median (Q1, Q3)	60.0 (52.0, 66.0)	64.0 (57.0, 69.0)	62.0 (55.0, 68.0)
Age class, n (%)			
<65 years	55 (67.9)	47 (54.0)	102 (60.7)
65-75 years	26 (32.1)	39 (44.8)	65 (38.7)
>75 years	0	1 (1.1)	1 (0.6)
Body mass index (kg/m^2^), median (Q1, Q3)	27.2 (24.3, 29.8)	29.8 (26.8, 34.3)	28.4 (25.7, 32.5)
Body mass index class, n (%)			
<25 kg/m^2^	25 (30.9)	11 (12.6)	36 (21.4)
25 – <30 kg/m^2^	37 (45.7)	33 (37.9)	70 (41.7)
30–35 kg/m^2^	11 (13.6)	26 (29.9)	37 (22.0)
>35 kg/m^2^	8 (9.9)	17 (19.5)	25 (14.9)

*Q* Quartile, *THR* Total hip replacement, *TKR* Total knee replacement

Concomitant cardiovascular medications, which are frequently prescribed in the orthopedic population, were reported in 93 (55.4 %) treated patients. Altogether, 161 patients received both enoxaparin and dabigatran; 2 received dabigatran but not enoxaparin, and 5 received enoxaparin but not dabigatran. Concerning enoxaparin treatment, median number of days under enoxaparin treatment was 2.0 days. Of the patients receiving enoxaparin, 31.9 % were treated for ≥4 days, 18.1 % for ≥7 days, and 15.1 % for ≥10 days.

Of the 168 treated patients, 139 (85.3 %) patients completed dabigatran treatment as planned. Out of the remaining 29 patients, 5 did not start dabigatran, and 24 prematurely discontinued treatment (12 due to adverse events [AEs], 4 lost to follow-up, 2 refused to continue taking dabigatran, 4 switched to other anticoagulants, and 2 discontinued for other reasons).

Most treated patients (82.6 %) received their first oral dose of dabigatran 22 to <26 h after the last dose of enoxaparin, and most patients (88.7 %) received their first oral dose of dabigatran ≥24 h after the end of surgery (Table 2). The median time from last enoxaparin dose to first oral dose of dabigatran was 24 h (range: 4–48 h). Of patients treated with dabigatran, 84.7 % took dabigatran for ≥28 days. The median number of days under dabigatran treatment was 36 days which was comparable for THR and TKR patients (medians: 35.5 and 37 days, respectively).

**Table 2:** Patient characteristics related to first intake of dabigatran

	THR	TKR	Total
Treated	81	87	168
Time from last enoxaparin to first dose dabigatran (hours)			
N	79	82	161
Median (Q1, Q3)	24.0 (24.0, 24.0)	24.0 (24.0, 24.0)	24.0 (24.0, 24.0)
Minimum	9	4	4
Maximum	48	48	48
Time from last enoxaparin to first oral dose class (n [%])			
0 – <14 hours	2 (2.5)	6 (7.3)	8 (5.0)
14 – <22 hours	4 (5.1)	2 (2.4)	6 (3.7)
22 – <26 hours	65 (82.3)	59 (72.0)	124 (77.0)
≥26 hours	8 (10.1)	15 (18.3)	23 (14.3)
Missing	2 (2.5)	5 (5.7)	7 (4.2)
Time to first oral dose after end of surgery (hours)			
N	79	83	162
Median (Q1, Q3)	35.0 (31.3, 101.7)	33.6 (29.0, 99.3)	34.3 (29.8, 99.5)
Time to first oral dose after end of surgery class			
Prior to end of surgery	0	0	0
0 – <1 hours	1 (1.2)	0	1 (0.6)
1 – <4 hours	4 (4.9)	5 (5.7)	9 (5.4)
4 – <8 hours	0	3 (3.4)	3 (1.8)
8 – <24 hours	0	0	0
≥24 hours	74 (91.4)	75 (86.2)	149 (88.7)
Missing	2 (2.5)	4 (4.6)	6 (3.6)
Relationship of first dabigatran dose to date of discharge (n [%])			
1 or more days before discharge	68 (84.0)	67 (77.0)	135 (80.4)
On day of, or 1 day after, discharge	10 (12.3)	10 (11.5)	20 (11.9)
Missing	3 (3.7)	10 (11.5)	13 (7.7)

*Q* Quartile

There were no reported occurrences of symptomatic VTE or all-cause mortality at any time during the study, resulting in an estimated incidence for the composite efficacy endpoint of 0.0 %; the limited sample size resulted in a wide 95 % CI (0.00-2.24).

The primary safety variable was MBEs from last application of enoxaparin 40 mg until 24 h after last intake of dabigatran. During this period, only 1 major bleeding event occurred, and this was at the surgical site (hematoma, resulting in an estimated incidence of MBE of 0.61 % (95 % CI: 0.02-3.37). This MBE required treatment cessation following TKR, but did not lead to a significant drop in hemoglobin or require transfusion. Overall, very few bleeding events were reported. From last application of enoxaparin 40 mg until 24 h after last intake of dabigatran, minor bleeding events were reported in 3 additional patients. There were no major extrasurgical-site bleeding events at any time during the study. An additional minor bleeding event was reported in a patient during enoxaparin treatment

During treatment with dabigatran, 44/163 (27.0 %) patients experienced ≥1 reported AE; of those, 13 (8.0 %) patients had AEs leading to discontinuation of dabigatran. Four patients (2.5 %) experienced serious AEs during treatment with dabigatran (hepatic enzyme increase, renal failure, myocardial infarction, and device dislocation).

## Discussion

This study, performed in a “real-life” clinical setting, sought to evaluate the safety and efficacy of a switch from enoxaparin 40 mg qd to dabigatran 220 mg qd for prevention of VTE after elective hip or knee replacement surgery. The frequency and types of reported AEs, serious AEs, and AEs leading to discontinuation of dabigatran after switching from LMWH, were consistent with the known safety profile for dabigatran. The incidence of bleeding events was low and no symptomatic VTE occurred. The switch from enoxaparin to dabigatran was well tolerated. The study did not detect any new safety issues when switching from LMWH to dabigatran in patients undergoing elective orthopedic surgery. The AEs and serious AEs were within the ranges reported in earlier clinical trials with dabigatran. Thus, these observational data support the feasibility and safety of switching from enoxaparin to dabigatran.

The feasibility of switching from LMWH to NOAC is an important, clinically relevant question in orthopedic practice [4, 21, 25]. In the 9th edition of the American College of Clinical Pharmacology (ACCP) guidelines, NOACs have been included in the list of antithrombotic therapies to be considered for patients undergoing total hip arthroplasty or total knee arthroplasty surgery [4]. Although different guidelines exist regarding the prevention of VTE in orthopedic surgery, no consensus has been achieved on whether the optimal start of LMWH is pre-, peri- or postoperative. The preferred timing of initiation of thromboprophylaxis varies among countries and has been extensively debated [26]. A perioperative start is apparently more effective, but this is counterbalanced by a marked increase in the risk of major bleeding in comparison with a preoperative or postoperative regimen [21].

The procedure of switching from enoxaparin to dabigatran has been evaluated in healthy volunteers in an earlier study [27]. Prior administration of enoxaparin for 3 days did not meaningfully affect the pharmacokinetic or pharmacodynamic properties of dabigatran. The ratios of free versus total dabigatran were not changed by pretreatment with enoxaparin and no drug-related AEs, such as any bleeding or hematoma, were observed.

Clinical experience regarding switching has also been obtained in studies designed for the treatment of an acute VTE. In the phase 3 RE-COVER study, all subjects were initially treated with a parenteral anticoagulant, mostly LMWH, which was applied at a higher dose than for VTE prevention [24, 28]. Parenteral anticoagulation was administered for ≥5 days together with either warfarin or warfarin placebo, until the international normalized ratio was ≥2 at 2 consecutive measurements. The parenteral anticoagulant was then discontinued and patients either continued warfarin or were switched to dabigatran 150 mg twice daily. The first dabigatran dose was given 0–2 h before the time when the next dose of the parenteral therapy would have been due. In this study, during the 6-month treatment period, there were fewer major or clinically relevant nonmajor bleeding events and similar rates of MBEs in the dabigatran group compared with the warfarin group [24, 28]. This study demonstrated the efficacy and safety of switching to dabigatran 150 mg twice daily in this patient population, even after the shorter time frame of 12 h after the last dose of enoxaparin.

In addition, the safety of switching was investigated in patients with atrial fibrillation (RE-LY trial of dabigatran 150 or 110 mg twice daily versus warfarin), when treatment for surgery or a diagnostic procedure was interrupted temporarily by the investigators [29]. According to the study protocol, discontinuation of dabigatran for between 1 and 5 days was recommended depending on the type of procedure and the anticipated bleeding risk, and short-term bridging anticoagulant therapy (e.g., LMWH) was used, if appropriate. Temporary interruptions occurred in 4623 patients. Stroke, myocardial infarction, and bleeding events were similar in all treatment groups within 30 days of reinitiating study medication. The authors concluded that switching between a parenteral anticoagulant and dabigatran resulted in effective and safe management of patients.

Furthermore, a clinical trial investigating the effects of switching from enoxaparin to the new oral factor Xa inhibitor, rivaroxaban, in patients undergoing THR or TKR has been published [30]. In 56 patients, the authors found no cases of VTE or bleeding, and no unexpected AEs, if rivaroxaban was initiated 12 or 24 h after the last LMWH dose.

Current prescribing information for dabigatran recommends that the anticoagulant should be initiated orally within 1–4 h of completed surgery if hemostasis has been obtained. This regimen has been successfully applied in the RE-NOVATE, RE-NOVATE II and RE-MODEL studies [16–18]. However, this is not always possible. In this present study, anticoagulation was started with LMWH and the process of switching to dabigatran was left to the decision of the treating physician. The median time of the first dabigatran tablet was 24.0 h after the last LMWH dosage and nearly three-quarters of patients got the first dabigatran dosage between 22 h and 26 h after last LMWH dosage. While some delays in therapy were due to clinical reasons (bleeding, drainage, or vomiting), the majority were for logistical reasons, including late-day surgery, organization of the ward, and nursing error. If initiation of dabigatran therapy is delayed, it can be started with a half-dose >4 h postsurgery if therapy begins on the same day as surgery, or, with the full dose the day after surgery. Eriksson BI, et al. investigated the effect of delaying the initiation of oral therapy with dabigatran and found that the efficacy in patients with a delay in the administration of the 220 mg qd dose was similar to that in patients without delayed dosing, even if the dose was delayed until the day after surgery [31].

## Conclusions

This study addressed the feasibility of switching from the LMWH, enoxaparin, to oral therapy with dabigatran, and closed a gap that has not been investigated in detail in phase 3/4 clinical trials for dabigatran in orthopedic surgery. The switching procedure did not raise any new safety issues or efficacy problems. The observed AEs were consistent with the known safety profile of dabigatran.

## Abbreviations

ACCP: American College of Clinical Pharmacology
AE: Adverse event
CI: Confidence interval
DVT: Deep vein thrombosis
LMWH: Low molecular weight heparin
MBE: Major bleeding event
NOAC: non-vitamin K antagonist oral anticoagulant
PE: Pulmonary embolism
qd: Once daily
THR: Total hip replacement
TKR: Total knee replacement
VTE: Venous thromboembolytic event
